# Prognostic Analysis of Endoprosthetic Reconstruction Versus Biological Reconstruction in the Treatment of Extremity Osteosarcoma

**DOI:** 10.3390/cancers18040610

**Published:** 2026-02-13

**Authors:** Guoxin Qu, Shengbiao Ma, Zhehuang Li, Zhichao Tian, Jiaqiang Wang, Xin Wang, Peng Zhang, Xiaohui Niu, Weitao Yao

**Affiliations:** 1 Department of Bone and Soft Tissue Cancer, The Affiliated Cancer Hospital of Zhengzhou University & Henan Cancer Hospital, Dongming Road 127, Zhengzhou 450008, China; zlyyqgx4259@zzu.edu.cn (G.Q.); jrqxnha_m@163.com (S.M.); zlyylizhehuang4413@zzu.edu.cn (Z.L.); tianzhichaoyy@163.com (Z.T.); doctorwangjiaqiang@126.com (J.W.); superwx1984@163.com (X.W.); zdzp@zzu.edu.cn (P.Z.); 2 Department of Orthopedic Oncology Surgery, Beijing Ji Shui Tan Hospital, 31 Xinjiekou East Street, Xicheng District, Beijing 100035, China; niuxiaohui@263.net

**Keywords:** biological reconstruction, liquid nitrogen devitalization, endoprosthetic, prognosis, osteosarcoma

## Abstract

**Objective**: To compare the clinical prognosis of metal endoprosthetic reconstruction versus biological reconstruction in the treatment of limb osteosarcoma and to analyze associated prognostic factors. **Methods**: From October 2014 to October 2021, a retrospective study was carried out of patients with high-grade extremity osteosarcoma. Patients were categorized into two groups based on the type of reconstruction: endoprosthetic reconstruction and biological reconstruction. Demographic data and prognosis were systematically compared between the two groups. Furthermore, a Cox proportional hazards model was employed to evaluate the risk factors associated with recurrence and survival outcomes. **Results**: A total of 133 patients were enrolled in the study, comprising 88 patients in the endoprosthetic reconstruction group and 45 patients in the biological reconstruction group. The 5-year overall survival (OS) and disease-free survival (DFS) rates for the endoprosthetic reconstruction group were 76.2% and 70.5%, respectively, which were higher than those observed in the biological reconstruction group (64.3% and 60%). Additionally, the local recurrence rate was significantly higher in the biological reconstruction group compared to the endoprosthetic reconstruction group (17.8% vs. 2.3%, *p* = 0.004). Cox regression analysis revealed that pathological fracture (*p* = 0.034) and the biological reconstruction (*p* = 0.007) were independent risk factors for local recurrence. **Conclusions**: Endoprosthetic reconstruction may be preferable for patients requiring early functional recovery or presenting with pathological fractures. Biological reconstruction may be considered for younger patients with diaphyseal defects and demanding long-term functional requirements, albeit with elevated local recurrence risk. Individualized decision-making incorporating tumor location, patient age, and functional goals is essential.

## Simple Summary

Osteosarcoma is a rare primary malignant bone tumor. Biological reconstruction techniques for bone defects following tumor resection remain less frequently adopted globally compared to endoprosthetic approaches. This retrospective study compared oncologic outcomes between endoprosthetic and biological reconstruction in 133 extremity osteosarcoma patients. Results indicate that endoprosthetic reconstruction was associated with superior local control and survival metrics, providing evidence-based guidance for surgical strategy selection in clinical practice.

## 1. Background

Osteosarcoma is the most common primary malignant bone tumor, with an incidence rate of approximately 2–3 cases per million individuals annually. It predominantly affects adolescents and arises in the metaphyseal region of long bones [1]. Over the past three decades, multimodal therapy—including neoadjuvant/adjuvant chemotherapy and refined surgical techniques—has significantly improved survival and limb salvage rates [2]. Due to the inherent radioresistance, wide-margin surgical resection remains the cornerstone of local control [1]. However, reconstruction of critical-sized bone defects post-resection presents ongoing clinical challenges. Current strategies include biological approaches (massive allograft transplantation, autograft devitalization and replantation, vascularized/non-vascularized fibular grafting) and endoprosthetic techniques (conventional modular prostheses, patient-specific 3D-printed implants) [3,4,5,6]. Biological reconstruction preserves native bone biology, avoids long-term prosthetic complications (e.g., aseptic loosening, wear), and supports potential growth in skeletally immature patients. However, prolonged consolidation periods necessitate extended non-weight-bearing protocols, resulting in suboptimal early function [7]. Conversely, endoprosthetic reconstruction provides immediate mechanical stability, facilitates early mobilization and weight-bearing, yet carries risks of long-term mechanical failure, infection, and revision surgery—particularly concerning in pediatric populations requiring multiple future revisions [8,9,10].

Massive allografts face limitations including donor scarcity, immunogenicity, nonunion, and poor osseointegration [11]. Autograft devitalization and replantation circumvents graft availability constraints. Among devitalization methods, liquid nitrogen treatment offers a cost-effective, technically accessible option with growing global adoption [12,13]. Due to the suboptimal outcomes associated with autograft inactivation and replantation in joint-related cases, early complications such as joint collapse and fractures may arise. Consequently, autograft inactivation and replantation are predominantly employed for the reconstruction of diaphyseal defects that do not involve the articular surface [14]. For bone defects involving joints, the primary reconstruction approach remains metal joint prostheses. In cases of diaphyseal defects, 3D-printed metal prostheses can also be utilized for reconstruction. To date, favorable outcomes have been observed in both the short and medium terms; however, long-term efficacy still requires further follow-up evaluation [15,16].

Currently, our hospital extensively employs endoprosthetic reconstruction and biological reconstruction via replantation with liquid nitrogen devitalization. This study aims to analyze the tumor control and survival outcomes of patients who underwent biological reconstruction or endoprosthetic reconstruction through medium- and long-term follow-up data. Additionally, it seeks to compare the differences between these two methods, thereby providing valuable references for selecting appropriate treatment strategies for patients.

## 2. Materials and Methods

We conducted a retrospective analysis of osteosarcoma patients who underwent limb-salvage surgery at our institution between October 2014 and October 2021. The inclusion criteria were as follows: (1) pathologically confirmed high-grade osteosarcoma of the extremities; (2) all patients received neoadjuvant and adjuvant chemotherapy; (3) reconstruction was performed using either liquid nitrogen-inactivated replantation or endoprosthetic techniques; (4) follow-up continued until death or exceeded three years. The exclusion criteria were: (1) patients with newly diagnosed metastatic disease; (2) intraoperative R1 or R2 resection, or postoperative pathological findings indicating positive margins; (3) patients lost to follow-up. All patients voluntarily provided written informed consent to participate in this study. For underage patients, the informed consent form was expressly signed by their legal guardians on their behalf.

The pathological types of all patients were confirmed via biopsy following the completion of relevant imaging examinations. Subsequently, two cycles of neoadjuvant chemotherapy were administered using either the MAP regimen (Doxorubicin, Cisplatin, and high-dose Methotrexate) or the AP regimen (Doxorubicin and Cisplatin) [17]. After neoadjuvant chemotherapy, the tumor was re-evaluated using imaging techniques. The surgical plan was discussed with the patient as follows: (1) Amputation was recommended for patients who were not candidates for limb salvage surgery; (2) For patients with redeemable limbs, liquid nitrogen inactivated replantation or metal endoprosthetic replacement was selected based on the patient’s preferences and the specific tumor conditions. Liquid nitrogen inactivated replantation was primarily employed in the reconstruction of diaphyseal bone defects that do not involve the articular surface. Nevertheless, six adult patients opted for 3D-printed metallic prostheses to meet the requirement of early postoperative mobilization. For those with tumors located adjacent to the articular surface, two cases underwent liquid nitrogen inactivated autograft reconstruction due to financial limitations, whereas the remaining patients received prosthetic joint replacement. Postoperative chemotherapy was continued for four cycles. All treatment regimens were finalized following multidisciplinary team (MDT) discussions.

The extent of surgical resection was determined based on preoperative magnetic resonance imaging (MRI). The tumor and adjacent normal tissue were excised en bloc, with a minimum margin of 2 cm. The minimum margin for epiphyseal osteotomy patients is 1 cm. Liquid nitrogen devitalization protocol: Following soft tissue stripping, the tumor-bearing bone segment was irrigated with sterile saline, immersed in liquid nitrogen for 30 min, thawed at room temperature (24–26 °C) for 20 min, and rehydrated in distilled water for 15 min. The devitalized autograft was then reimplanted and stabilized with a locking plate. In select cases, non-vascularized autogenous or allogeneic fibular strut grafts were employed as a mesenchymal graft to enhance the structural integrity of the inactivated bone.

For biological reconstruction, non-weight-bearing mobilization commenced immediately postoperatively; partial weight-bearing was initiated at approximately 3 months, with full weight-bearing permitted only after radiographic confirmation of solid union. For endoprosthetic cases, progressive weight-bearing began after drain removal, typically within 48–72 h, and early full weight-bearing may be considered depending on the clinical scenario.

The fundamental characteristics of all included patients were retrospectively analyzed. Patients underwent follow-up evaluations every three months during the first three years, every six months during the fourth and fifth years, and annually after five years. These follow-ups were conducted to monitor for tumor recurrence and metastasis, as well as to assess bone healing and the status of prostheses.

## 3. Statistical Analysis

The Kaplan–Meier method was employed to estimate and compare overall survival (OS) and disease-free survival (DFS) rates. Cox proportional hazards model was utilized to evaluate potential risk factors. Categorical variables were compared using the chi-square test, while continuous variables were analyzed using the independent t-test. All statistical analyses were conducted using SPSS version 29.0 (IBM Corporation, Armonk, NY, USA) and R version 4.43 (R Development Core Team, 2010, Vienna, Austria). A two-sided *p*-value of less than 0.05 was considered statistically significant.

## 4. Results

### 4.1. General Data

A total of 133 patients who met the inclusion and exclusion criteria were recruited, comprising 81 males and 52 females. Among them, 45 patients were assigned to the biological reconstruction group, with a mean age of 16 years. In contrast, 88 patients were allocated to the endoprosthetic reconstruction group, with a mean age of 17.5 years. Notably, 14 patients experienced pathological fractures, including 6 in the biological reconstruction group and 8 in the endoprosthetic reconstruction group. The mean ages of the two groups were statistically significantly different (*p* = 0.042), with the biological reconstruction group being younger than the endoprosthetic reconstruction group. No significant differences were observed between the two groups regarding gender distribution, body mass index (BMI), tumor diameter, or extent of bone involvement. In the biological reconstruction group, the tumor locations were distributed as follows: femur in 23 cases, tibia in 13 cases, humerus in 4 cases, tibia with fibular invasion in 3 cases, and ulna/radius in 2 cases. In the prosthetic reconstruction group, the distribution was as follows: femur in 52 cases, tibia in 32 cases, and humerus in 4 cases. There was no statistically significant difference between the two groups in the common sites, while there was a statistically significant difference between the groups in the rare sites (*p* = 0.004). (Table 1).

### 4.2. Oncological Outcomes

**Figure 1:**
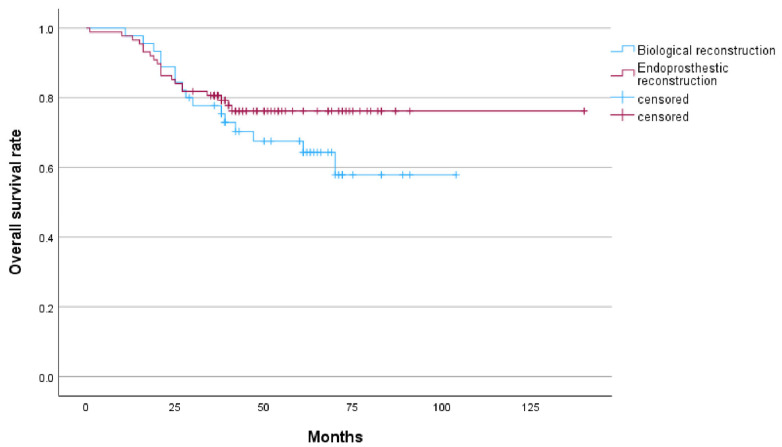
Kaplan–Meier curve showed the overall survival of endoprosthetic reconstrucion and biological reconstrucion.

**Figure 2:**
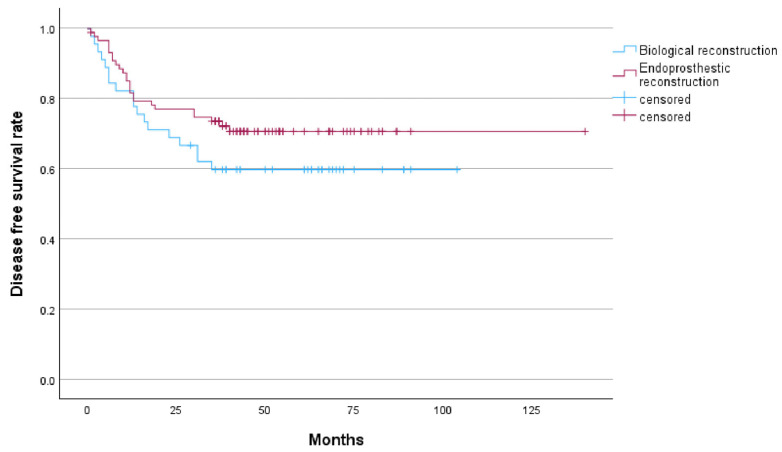
Kaplan–Meier curve showed the disease-free survival rate of endoprosthetic reconstrucion and biological reconstrucion.

In the biological reconstruction group, 18 cases (40%) experienced recurrence or metastasis, comprising 8 cases of local recurrence with metastasis and 10 cases of distant metastasis alone. All recurrences were located within the surrounding soft tissues. In the endoprosthestic reconstruction group, 25 cases (28.4%) experienced recurrence or metastasis, including 1 case of local recurrence that was successfully managed following amputation, 1 case of local recurrence with metastasis, and 23 cases of distant metastasis. The 3-year OS and 5-year OS rates in the biological reconstruction group were 77.7% and 64.3%, respectively. The corresponding 3-year DFS and 5-year DFS rates were 60% each. In the endoprosthetic reconstruction group, the 3-year OS and 5-year OS rates were 80.7% and 76.2%, respectively, while the 3-year DFS rate was 72.2% and the 5-year DFS rate was 70.5%. Recurrence occurred in 8 cases (17.8%) in the biological reconstruction group and 2 patients (2.3%) in the endoprosthetic reconstruction group, with a statistically significant difference (*p* = 0.004). Among the recurrent cases in the biological reconstruction group, prior to treatment, two cases presented with pathological fractures, one case exhibited a skipping lesion of the femur, and one case involved unplanned internal fixation. Additionally, patients in both groups who experienced recurrence exhibited large soft tissue masses. Among the 36 deceased patients across both groups, 35 succumbed to tumor-related factors, while 1 patient in the endoprosthestic reconstruction group died due to bone marrow suppression complications from postoperative adjuvant chemotherapy. (Table 2, Figure 1 and Figure 2).

**Figure 3:**
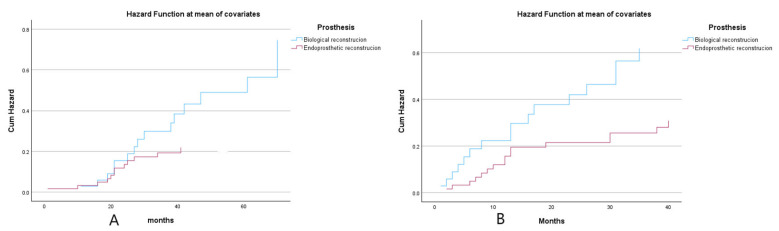
(**A**): Age-adjusted hazard ratio for overall survival rate of endoprosthetic reconstrucion and biological reconstrucion. (**B**): Age-adjusted hazard ratio for disease-free survival rate of endoprosthetic reconstrucion and biological reconstrucion.

Further subgroup analysis was conducted specifically for pediatric and adolescent patients within both groups. In the biological reconstruction group comprising 35 patients, the mean age was 12.1 ± 2.5 years, while the endoprosthetic reconstruction group included 63 patients with a mean age of 13.4 ± 2.9 years. A statistically significant difference in age was observed between the two groups (*p* = 0.025). Age-adjusted analysis revealed that biological reconstruction was associated with elevated risks of mortality and disease recurrence with increasing survival duration. (Figure 3).

Cox proportional hazards model analysis revealed that pathological fracture (*p* = 0.034) and biological reconstruction (*p* = 0.07) were associated with an increased risk of local recurrence (*p* < 0.05). Although pathological fracture (*p* = 0.073) and tumor diameter (*p* = 0.066) did not reach statistical significance for OS, their *p*-values were close to the threshold of 0.05, suggesting a potential influence of these factors on survival outcomes. Other variables, including age and bone involvement, showed no significant association with either local recurrence or survival. Additionally, no statistically significant factors were identified in relation to DFS. (Table 3, Table 4 and Table 5).

**Figure 4:**
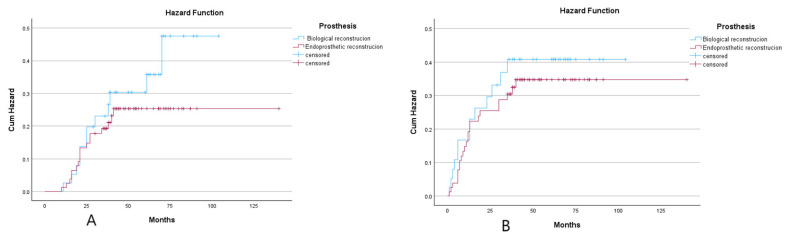
(**A**): The hazard ratio for overall survival rate after excluding patients with pathological fractures in the biological reconstruction group versus the prosthetic reconstruction group. (**B**): Hazard ratio for disease-free survival rate after excluding patients with pathological fractures in the biological reconstruction group versus the prosthetic reconstruction group.

Given the significant impact of pathological fractures on recurrence and metastasis, we excluded patients with such fractures and conducted a subgroup analysis of the remaining 119 patients. The results revealed that biological reconstruction remained associated with higher tumor recurrence rates and poorer overall survival. (Figure 4).

### 4.3. Complications

Infection was observed in 3 cases (6.7%) of patients undergoing biological reconstruction treatment, and all cases were cured following thorough debridement and antibiotic therapy. Among the 7 cases (7.9%) of patients who underwent endoprosthesis reconstruction, 4 cases presented with superficial wound infections and showed improvement after simple debridement and antibiotic therapy. Three cases (3.4%) of periprosthetic infection were reported. One case of proximal tibia had a deep—incision infection and was cured after flap transplantation. One patient with proximal tibia underwent amputation due to prosthesis infection. Another patient with distal femur experienced postoperative prosthesis infection complicated by osteomyelitis, developed bone marrow suppression after chemotherapy, and ultimately died of septic shock.

**Figure 5:**
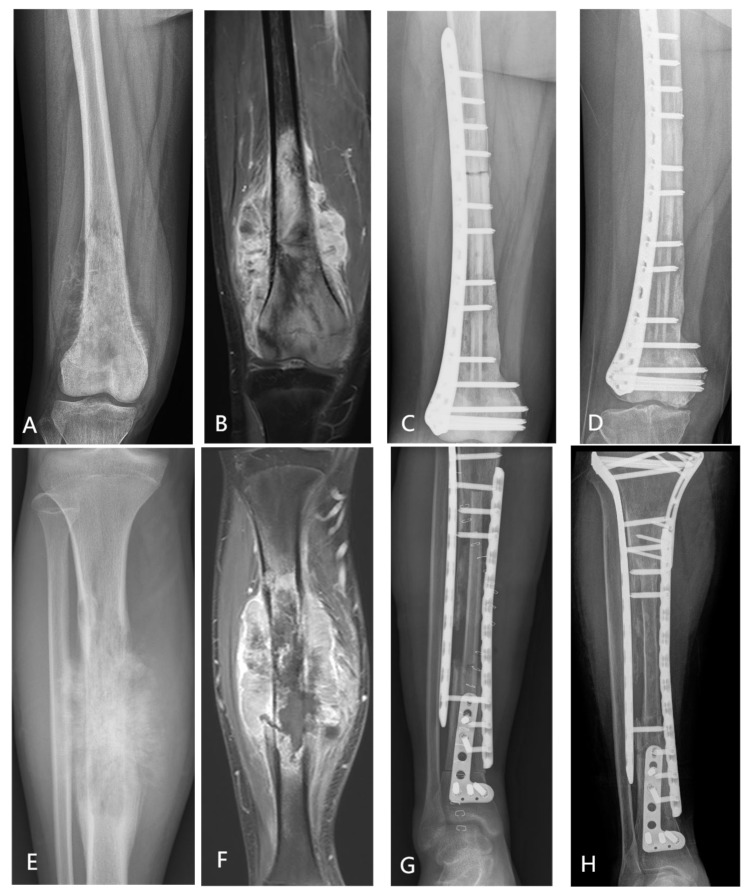

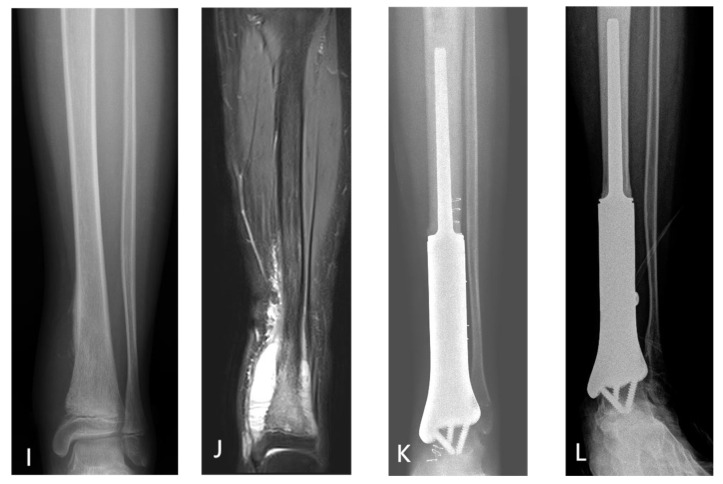
(**A**–**D**) A 15-year-old female with osteosarcoma in the right femur underwent biological reconstruction using liquid nitrogen inactivated replantation combined with an allogeneic fibular graft. (**A**,**B**) X-ray and MRI showed the tumor involvement in the right femoral. (**C**) Immediate postoperative X-ray image. (**D**) Five-year postoperative X-ray image. (**E**–**H**) A 17-year-old male with osteosarcoma in the right tibia underwent biological reconstruction using liquid nitrogen inactivated replantation combined with autologous fibular graft. (**E**,**F**) X-ray and MRI showed the tumor involvement in the right tibia. (**G**) Immediate postoperative X-ray image. (**H**) Five-year postoperative X-ray image. (**I**–**L**) A 12-year-old male diagnosed with osteosarcoma in the left tibia underwent reconstruction using a 3D-printed prosthetic implant.

Among the 45 patients who underwent biological reconstruction, 31 patients received epiphyseal osteotomy with joint preservation, and 14 patients underwent osteotomy without joint preservation. Primary bony union was achieved at 45.2% (14/31) of epiphyseal osteotomy sites and at 35.6% (16/45) of diaphyseal osteotomy sites. The mean time to bone healing differed significantly between epiphyseal (13.8 ± 6.4 months) and diaphyseal (25.8 ± 8.6 months) osteotomy sites (*p* = 0.001). All four patients who received humeral inactivated autologous tumor bone replantation demonstrated radiographically confirmed progressive bone resorption. Multivariate logistic regression analysis identified both tumor location and osteotomy site (metaphyseal vs. diaphyseal) as independent predictors of primary bony union (both *p* < 0.05), whereas osteotomy length was not significantly associated with union outcome (*p* = 0.52). Figure 5 illustrates three representative surgical cases. (Figure 5).

## 5. Discussion

Currently, the predominant method for reconstructing local bone defects in osteosarcoma remains endoprosthetic reconstruction. While endoprosthetic reconstruction offers immediate stability and enables early weight-bearing for patients, it is associated with long-term complications such as prosthesis loosening, fractures, wear, and infections, among others [8,18]. Especially for adolescent patients, multiple prosthesis revision surgeries are frequently required. In recent years, biological reconstruction methods, such as liquid nitrogen inactivated replantation, have gained increasing attention and application. This is because these methods can effectively avoid long-term complications associated with prostheses and offer greater potential advantages, particularly for pediatric and adolescent populations [19,20]. However, biological reconstruction frequently encounters the risks of transient suboptimal function, bone nonunion, bone resorption, and fracture [11,14,21]. Pathological fractures have been regarded as a contraindication for inactivated replantation. However, in this study, owing to the limited development of early-stage 3D-printed metal prosthesis technology and financial limitations faced by certain patients, the inactivated replantation technique was ultimately selected for six cases of pathological fractures. In patients with pathological fractures, the local recurrence rate following biological reconstruction can be as high as 33% (2/6), whereas no recurrence has been observed in endoprosthetic reconstruction cases up to the follow-up cutoff date. This finding suggests that, for patients with pathological fractures who face financial constraints and cannot afford prosthetic replacement, amputation represents a viable alternative [22]. The younger age in the biological reconstruction group (mean 15.96 years vs. 17.51 years, *p* = 0.042) may reflect a clinical preference for bioreconstruction in children and adolescents to preserve growth potential and better long-term function. However, the biological reconstruction group has a longer bone healing time and poor short-term functional recovery, which may affect the quality of life and compliance of patients with subsequent treatment.

Funovics et al. previously reported that no significant difference was observed in the prognosis of patients with parosteal osteosarcoma treated with either biological reconstruction or endoprosthetic reconstruction [23]. However, in high-grade osteosarcoma cases, the biological reconstruction group exhibited a significantly higher local recurrence rate compared to the endoprosthetic group (17.8% vs. 2.3%, *p* = 0.004). This difference may be attributed to microenvironmental alterations during the healing process between the killed bone and host bone, as well as patient selection criteria. Cox regression analysis indicated that both pathologic fracture (*p* = 0.034) and biological reconstruction (*p* = 0.007) serve as independent risk factors for local recurrence. Pathologic fractures might cause local dissemination of tumor cells and compromise the accuracy of surgical margin assessment, thereby contributing to an elevated recurrence rate [24]. Among the 8 cases with recurrence in the biological reconstruction group, two experienced pathological fractures, one underwent unplanned surgery, and one exhibited skip lesions, all of which may contribute to an elevated local recurrence rate. In addition, Patients undergoing biological reconstruction are typically younger with underdeveloped peri-tumoral musculature and soft tissues. This may compromise the assessment of imaging studies and establishment of an adequate soft tissue barrier during surgery compared to adults, which could contribute to a higher risk of recurrence and metastasis [25]. Additionally, in joint-sparing epiphyseal osteotomy, some studies suggest that a 1 cm margin from the tumor may suffice as a safe surgical boundary [26]. While our institution also adopts this approach, it may entail potential risks of insufficient surgical margins, thereby elevating the likelihood of recurrence and metastasis following biological reconstruction. Achieving adequate surgical exposure during joint preservation procedures tends to be more challenging. These factors may collectively contribute to an increased risk of local recurrence [27]. In contrast, for patients undergoing endoprosthetic reconstruction, we consistently achieve an effective osteotomy length with a 2 cm extended margin. At the same time, we observed that the remaining recurrent patients in both groups typically presented with large soft tissue masses. This indicates that for patients with extensive soft tissue masses, amputation may be preferable to achieve a safer surgical margin. Additionally, for patients with pathological fractures, endoprosthetic reconstruction or amputation is recommended as appropriate treatment options.

There was no significant difference in survival outcomes between the two groups. Cox regression confirmed pathological fracture and biological reconstruction as independent predictors of local recurrence. Although reconstruction type did not reach statistical significance for OS in multivariate analysis (HR = 0.64, *p* = 0.179), the endoprosthetic group demonstrated numerically higher 5-year OS (76.2% vs. 64.3%) and DFS (70.5% vs. 60.0%), suggesting potential indirect benefits of early functional recovery on overall treatment tolerance and outcomes [27,28]. Further Cox regression analysis revealed that the *p*-values for pathological fracture and tumor diameter with respect to OS were close to the significance threshold (*p* ≈ 0.05), indicating that these factors may exert a certain influence on survival. Pathological fractures and larger tumor volumes were associated with an elevated risk of local soft tissue invasion and hematogenous metastasis, which contributed to poorer prognoses [29,30]. However, the extent of bone invasion did not have a statistically significant impact on tumor recurrence or metastasis, suggesting that the primary prognostic factors for osteosarcoma are tumor volume and soft tissue margins [31,32].

This study revealed that the primary factors influencing bone healing in biological reconstruction were the tumor site and the osteotomy location, which was consistent with previous findings from our center [33]. Our study indicated that tumor length exerts a certain influence on patient survival, with larger tumor correlating with poorer prognosis (*p* = 0.06). However, no statistically significant difference was observed in terms of local recurrence (*p* = 0.95). Concurrently, it was found that the resection length of the tumor did not significantly affect bone healing, infection rates, or functional outcomes. The principal determinant of postoperative function lied in the preservation of tendon attachment sites at the articular region. The incidence of infection was primarily influenced by tumor location and the method of reconstruction, with endoprosthetic reconstruction associated with a heightened risk of refractory infections.

This study had certain limitations. First, a retrospective study design may introduce selection bias, particularly among younger patients who were more inclined to opt for biological reconstruction. Second, the sample size was limited. The total cohort and number of local recurrence events are relatively small, potentially limiting the robustness of Cox regression results. Third, In this study, some patients who underwent biological reconstruction received supplemental autogenous or allogenous fibular segments due to insufficient mechanical strength of the inactivated bone, which may have contributed to heterogeneity in the outcomes. Additionally, the use of bespoke 3D-printed prostheses in selected cases with unique anatomical configurations might have exerted a potential influence on the results. Future prospective multicenter studies are necessary to further control for confounding factors and larger samples to validate the conclusions.

## 6. Conclusions

Biological reconstruction is best suited for skeletally immature patients with diaphyseal defects where growth preservation and long-term biological integration are prioritized. However, the elevated local recurrence risk necessitates stringent patient selection, meticulous surgical margins, and vigilant surveillance. Conversely, endoprosthetic reconstruction offers superior local control and rapid functional restoration, particularly advantageous in pathological fracture scenarios or when early ambulation is critical. The presence of large soft tissue masses warrants careful margin assessment; amputation may be considered when achieving wide margins is unfeasible. Clinical decision-making should take into account the anatomical location of the tumor, the patient’s age, and specific functional requirements to develop personalized treatment plans [4,34,35]. With advancing biomaterial science and additive manufacturing, next-generation patient-specific prostheses and optimized devitalization protocols hold promise for enhancing both oncologic safety and functional longevity. Future prospective, multicenter studies with larger cohorts are warranted to validate these findings and refine personalized reconstruction algorithms [5,36].

## Figures and Tables

**Table 1:** Patient demographic data of the two groups.

Characteristic	Biological Reconstruction (*n* = 45)	Endoprosthetic Reconstruction (*n* = 88)	*p*-Value
Sex, *n* (%)			0.681
female	16 (35.6%)	36 (40.9%)	
Male	29 (64.4%)	52 (59.1%)	
Mean age, yrs ± SD	15.96 ± 9.86	17.51 ± 8.37	0.042
Age group, *n* (%)			0.092
≤8 years	4 (8.9%)	2 (2.3%)	
8–15 years	28 (62.2%)	48 (54.5%)	
>15 years	13 (28.9%)	38 (43.2%)	
BMI ± SD	20.52 (±4.06)	20.77 (±4.03)	0.712
Location of tumor			
Femur	23 (51.1%)	52 (59.1%)	0.460
Tibia	13 (28.9%)	32 (36.4%)	0.446
Humerus	4 (8.9%)	4 (4.5%)	0.442
Fibula	3 (6.7%)	0	0.004
Ulna and radius	2 (4.4%)	0	
Diameter of tumor, *n* (%)			0.299
≤8 cm	26 (57.8%)	41 (46.6%)	
>8 cm	19 (42.2%)	47 (53.4%)	
The extent of bone invasion, *n* (%)			0.985
<1/3 involved bone	31 (68.9%)	59 (67.0%)	
>1/3 involved bone	14 (31.1%)	29 (33.0%)	
Fracture, *n* (%)	6 (13.3%)	8 (9.1%)	0.649

**Table 2:** Prognosis of patient.

Prognosis	Biological Reconstruction (*n* = 45)	Endoprosthetic Reconstruction (*n* = 88) *	*p*-Value
Metastasis, *n* (%)	18 (40.0%)	24 (27.3%)	0.195
Local recurrence, *n* (%)	8 (17.8%)	2 (2.3%)	0.004
Survival state			0.171
Alive, *n* (%)	29 (64.4%)	68 (77.3%)	
Dead, *n* (%)	16 (35.6%)	20 (22.7%)	
3 years OS, *n* (%)	35 (77.7%)	71 (80.7%)	0.991
5 years OS, *n* (%)	29 (64.3%)	67 (76.2%)	0.126
3 years DFS, *n* (%)	27 (60.0%)	64 (72.2%)	0.15
5 years DFS, *n* (%)	27 (60.0%)	63 (70.5%)	0.248

* Of the 20 patients who died, 19 died of tumor factors and 1 died of chemotherapy-related complications 1 month after surgery.

**Table 3:** Cox of local recurrence.

Factor	All	HR	*p*-Value
Age, mean ± SD	17.0 ± 8.9	0.92 (0.81–1.04)	0.189
Diameter of tumor, *n* (%)		0.96 (0.28–3.33)	0.954
≤8 cm	67 (50.4%)		
>8 cm	66 (49.6%)		
The extent of bone invasion, *n* (%)		0.87 (0.22–3.35)	0.834
<1/3 involved bone	90 (67.7%)		
>1/3 involved bone	43 (32.3%)		
Fracture, *n* (%)		4.32 (1.12–16.7)	0.034
No	119 (89.5%)		
Yes	14 (10.5%)		
Reconstruction		0.12 (0.03–0.56)	0.007
Biological reconstruction, *n* (%)	45 (33.8%)		
endoprosthetic reconstruction, *n* (%)	88 (66.2%)		

*n* = 133 (represents the total number of cases), events = 10 (indicates the number of occurrences of the event in this Cox analysis table).

**Table 4:** Cox of DFS.

Factor	All	HR	*p*-Value
Diameter of tumor, *n* (%)		0.71 (0.39–1.29)	0.261
≤8 cm	67 (50.4%)		
>8 cm	66 (49.6%)		
The extent of bone invasion, *n* (%)		1.06 (0.56–2.01)	0.849
<1/3 involved bone	90 (67.7%)		
>1/3 involved bone	43 (32.3%)		
Fracture, *n* (%)		1.84 (0.82–4.13)	0.141
No	119 (89.5%)		
Yes	14 (10.5%)		
Reconstruction		0.66 (0.36–1.21)	0.178
Biological reconstruction, *n* (%)	45 (33.8%)		
endoprosthetic reconstruction, *n* (%)	88 (66.2%)		

*n* = 133 (represents the total number of cases), events = 43 (indicates the number of occurrences of the event in this Cox analysis table).

**Table 5:** Cox of OS.

Factor	All	HR	*p*-Value
Diameter of tumor, *n* (%)		0.53 (0.27–1.04)	0.066
≤8 cm	67 (50.4%)		
>8 cm	66 (49.6%)		
The extent of bone invasion, *n* (%)		0.81 (0.39–1.68)	0.571
<1/3 involved bone	90 (67.7%)		
>1/3 involved bone	43 (32.3%)		
Fracture, *n* (%)		2.13 (0.93–4.87)	0.073
No	119 (89.5%)		
Yes	14 (10.5%)		
Reconstruction		0.64 (0.33–1.23)	0.179
Biological reconstruction, *n* (%)	45 (33.8%)		
endoprosthetic reconstruction, *n* (%)	88 (66.2%)		

*n* = 133 (represents the total number of cases), events = 36 (indicates the number of occurrences of the event in this Cox analysis table).

## Data Availability

The datasets generated during and/or analysed during the current study are available from the corresponding author on reasonable request.

## Abbreviations

The following abbreviations are used in this manuscript:

OS	overall survival
DFS	disease-free survival
